# Downregulation of MYBL1 in endothelial cells contributes to atherosclerosis by repressing PLEKHM1-inducing autophagy

**DOI:** 10.1007/s10565-024-09873-6

**Published:** 2024-05-27

**Authors:** Shi-Ao Ding, Hao Liu, Rui Zheng, Yang Ge, Zheng Fu, Ju Mei, Min Tang

**Affiliations:** 1 Department of Cardiothoracic Surgery, Xinhua Hospital Affiliated to Shanghai Jiao Tong University School of Medicine, 1665 Kongjiang Road, Yangpu District, Shanghai, China; 2 Department of Pediatric Cardiovascular Surgery, Xinhua Hospital Affiliated to Shanghai Jiao Tong University School of Medicine, Shanghai, China

**Keywords:** Atherosclerosis, MYB proto-oncogene-like 1, Autophagy, Pleckstrin Homology and RUN domain containing M1

## Abstract

MYBL1 is a strong transcriptional activator involved in the cell signaling. However, there is no systematic study on the role of MYBL1 in atherosclerosis. The aim of this study is to elucidate the role and mechanism of MYBL1 in atherosclerosis. GSE28829, GSE43292 and GSE41571 were downloaded from NCBI for differentially expressed analysis. The expression levels of MYBL1 in atherosclerotic plaque tissue and normal vessels were detected by qRT-PCR, Western blot and Immunohistochemistry. Transwell and CCK-8 were used to detect the migration and proliferation of HUVECs after silencing MYBL1. RNA-seq, Western blot, qRT-PCR, Luciferase reporter system, Immunofluorescence, Flow cytometry, ChIP and CO-IP were used to study the role and mechanism of MYBL1 in atherosclerosis. The microarray data of GSE28829, GSE43292, and GSE41571 were analyzed and intersected, and then MYBL1 were verified. MYBL1 was down-regulated in atherosclerotic plaque tissue. After silencing of MYBL1, HUVECs were damaged, and their migration and proliferation abilities were weakened. Overexpression of MYBL1 significantly enhanced the migration and proliferation of HUVECs. MYBL1 knockdown induced abnormal autophagy in HUVEC cells, suggesting that MYBL1 was involved in the regulation of HUVECs through autophagy. Mechanistic studies showed that MYBL1 knockdown inhibited autophagosome and lysosomal fusion in HUVECs by inhibiting PLEKHM1, thereby exacerbating atherosclerosis. Furthermore, MYBL1 was found to repress lipid accumulation in HUVECs after oxLDL treatment. MYBL1 knockdown in HUVECs was involved in atherosclerosis by inhibiting PLEKHM1-induced autophagy, which provided a novel target of therapy for atherosclerosis.

**Graphical abstract:**

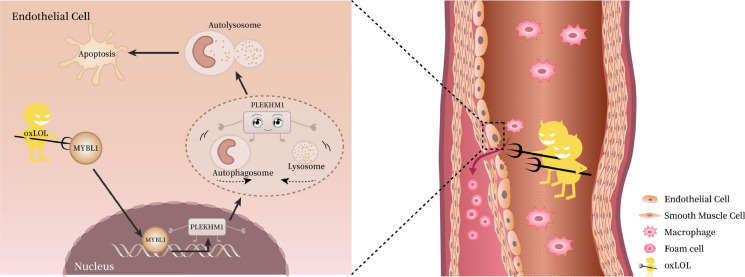

**Supplementary Information:**

The online version contains supplementary material available at 10.1007/s10565-024-09873-6.

## Introduction

As a chronic inflammatory disease, atherosclerosis (As) is the main cause of most cardiovascular diseases (CVD) worldwide. Its pathogenesis is complex and is the result of multiple factors. The main risk factors include hypertension, hyperlipidemia and heavy smoking and so on. Atherosclerosis is characterized by the formation of atherosclerotic plaques in the aorta, and its pathological basis is dysfunction of lipid metabolism. Endothelial cell injury and endothelial dysfunction play a key role in the development and progression of AS and are hallmarks of atherosclerosis (He et al. 2022; Liu et al. 2022; Zhang et al. 2022).

MYBL1 (MYB proto-oncogene like 1, MYBL1) is a member of MYB proto-oncogene family. It contains a conserved DNA binding homology domain and transcription activation domain, and is a strong transcription activator (Golay et al. 1994). MYBL1 is associated with a variety of diseases and participates in the malignant development of tumors. Brayer et al. revealed that repeated fusions of MYB and MYBL1 promoted the development of salivary adenoid cystic carcinoma by affecting the oncogenic pathway (Pei et al. 2019). Zhu et al. reported that MYBL1 could induce transcriptional activation of ANGPT2, which was closely related to higher endothelial vessel density, thereby affecting sorafenib resistance in hepatocellular carcinoma (Zhu et al. 2022). Nikolaus et al. suggested that MYBL1 could trigger autoimmune encephalitis and therefore play a role in disease immunity (Nikolaus et al. 2022). Yukari Endo et al. found that alterations in MYB and MYBL1 accelerated the development of early adenoid cystic carcinoma (Endo et al. 2019). However, there are few studies on the role of MYBL1 in atherosclerosis.

Autophagy can remove damaged organelles, misfolded proteins and other harmful substances through lysosomes, and achieve self-metabolism and renewal of cells (Xu et al. 2019; Fu et al. 2023; Alizadeh et al. 2023). It is an important mechanism for cells to maintain homeostasis. Pleckstrin homology domain containing protein family member 1 (PLEKHM1) is a communication bridge of autophagosome and lysosome, promoting autophagosome-lysosome fusion and associated with many diseases (McEwan et al. 2014). David G. McEwan et al. demonstrated that PLEKHM1 deletion caused a barrier to autophagic flow and impeded the autophagy-mediated degradation process, suggesting that PLEKHM1 might delay the development of atherosclerosis (McEwan et al. 2014). Permuth-Wey et al. found that the susceptibility locus for epithelial ovarian cancer contained PLEKHM1 and PLEKHM1 might mediate the initiation and progression of epithelial ovarian cancer (Permuth-Wey et al. 1627). Given the diverse effect of PLEKHM1 on different diseases, it is still unclear what role PLEKHM1 plays in the development of atherosclerosis.

Our study aims to clarify the mechanism of MYBL1 in endothelial cells involved in atherosclerosis by regulating PLEKHM1-induced autophagy, and to provide new understanding of atherosclerosis.

## Materials and methods

### Study design

The purpose of this study was to find a target of therapy for atherosclerosis. Firstly, through the intersection of three GEO arrays (GSE28829, GSE43292 and GSE41571) differential genes, we found a common target, MYBL1. Secondly, MYBL1 was knocked down or overexpressed in Human umbilical vein endothelial cells (HUVECs) to clarify the role of MYBL1. Thirdly, transcriptome sequencing was used to find the downstream signaling pathway of MYBL1. Finally, Western blot, immunofluorescence, LC3 track, Flow cytometry, ChIP and CO-IP were used to study the pathogenesis of MYBL1 in atherosclerosis.

### Tissue collection and cell culture

Human arterial tissues were obtained from patients undergoing dissection aneurysm surgery, and a total of 16 specimens were collected from December 10, 2018 to March 19, 2021. The sample size for the human were merely a convenience. HUVECs were obtained from ATCC (USA). 10% FBS (Gibco, Thermo Fischer Scientific, Bartlesville, OK, USA) and 60 µg/ml endothelial cell growth agent (BD Biosciences) were used to culture HUVECs.

### Animals and treatments

Seven-week-old male ApoE^−/−^ and ApoE^+/+^mice purchased from GemPharmatech Co., Ltd were used for the experiments. Two groups (ApoE^−/−^ and ApoE^+/+^ mice) fed a high-fat diets (0.15% cholesterol and 21% fat, Shanghai Medical Laboratory Animal Center) for 12 weeks. The environment was maintained on a 12-h light–dark cycle, and the mice had access to water AD libitum.

### Data

In order to study the expression changes of atherosclerotic plaque genes, the selected dataset must contain atherosclerotic plaque samples. The atherosclerosis expression profile datasets were obtained from the Gene Expression Omnibus (GEO) database, and the series of GSE28829 (https://www.ncbi.nlm.nih.gov/geo/query/acc.cgi?acc=GSE28829), GSE43292 (https://www.ncbi.nlm.nih.gov/geo/query/acc.cgi?acc=GSE43292), and GSE41571 (https://www.ncbi.nlm.nih.gov/geo/query/acc.cgi?acc=GSE41571) data were extracted for subsequent research and analysis. These samples all contained atherosclerotic plaque samples with different pfirrmann grades, and GSE28829 contained 13 atherosclerotic plaque samples (EA) and 16 advanced atherosclerotic plaque samples (AA). GSE43292 consisted of 32 specimens of early and advanced carotid atherosclerotic plaques. GSE41571 consisted of 5 samples of ruptured atherosclerotic plaques and 6 samples of stable atherosclerotic plaques.

### Small interfering RNA and Lentivirus transfection

Small interfering RNAs to MYBL1 (si-MYBL1, Gene Pharma, China) were mixed with Lipofectamine 3000 (Invitrogen, Carlsbad, California, USA) for 20 min in the serum-free medium and then added to HUVECs.

The LV3-pGLV-H1 + Puro plasmids with pcDNA-MYBL1, pcDNA-AMPK, pcDNA-GABARAP, pcDNA-PLEKHM1, shRNA-MYBL1 and shRNA-PLEKHM1 (Lenti-MYBL1, Lenti-PLEKHM1, Lenti-shMYBL1 and Lenti-shPLEKHM1) (Gene Pharma, China) were transfected into HUVECs following all manufacturer protocols.

### Differentially expressed genes identification

The probe names of GSE28829, GSE43292, and GSE41571 were converted into gene names. DESeq2, edgeR, and limma were used for differential analysis. Determination conditions for | log2 (FC) |> 1.5, adjusted *P*-value ≤ 0.05.

### Functional enrichment analysis

GO function (https://www.geneontology.org/) and KEGG (https://www.genome.jp/kegg/) pathway enrichment analysis was performed on differential genes, and important pathways were selected. Go enrichment analysis is a key tool to understand the molecular function of genes and the biological pathways involved. KEGG contains genome, chemistry, system function information and so on.

### Immunohistochemistry

The tissues were fixed with 4% formaldehyde solution (Beyotime, Shanghai, China) and subsequently dehydrated with high concentrations of ethanol (Damao, Tianjin, China), and the ethanol in the cells was displaced by the addition of xylene (Damao, Tianjin, China) for 30 min at room temperature. The tissues were embedded in paraffin for 3 h, then the tissues were cut into 4–5 μm and placed in constant temperature water at 40° C after the addition of 5% ethanol, followed by baking in an oven at 60° C for 30 min before removal for staining. Endogenous peroxidase and nonspecifically bound sites were blocked with 3% hydrogen peroxide and 5%BSA respectively. The primary antibody containing MYBL1 (Affinity, AF9007, 1:100) and PLEKHM1 (Abcam, ab204437, 1:50) was added to the sections and incubated in 10% goat serum blocking solution. The following day, the secondary antibody was adhered to the primary antibody and counterstained with hematoxylin.

### Transcriptome sequencing

Total RNA was extracted using TRIzol reagent (Invitrogen, Carlsbad, California, USA), followed by mRNA isolation using Nuceolspin RNA columns (Macherey–Nagel, Duren, Germany). cDNA was then synthesized using the Omniscript Reverse Transcription kit (Qiagen, UK). To smoothly build library and subsequent sequencing, FastQC (http://www.bioinformatics.babraham.ac.uk/projects/fastqc/) was used to detect the original read piece of raw reads the quality. After cDNA libraries were constructed, they were sequenced on Illumina HiSeq2500 V4 2 × 100 PE (Genewiz).

### Western blot

Extracted protein were obtained from tissues in atherosclerotic plaque tissue and HUVECs. Its concentration was tested with a BCA kit (Beyotime, Shanghai, China). The details refered to a previous study (Ding et al. 2020). The primary antibodies were listed as follow: MYBL1 (Affinity, AF9007, 1:1000), PLEKHM1 (Proteintech, 16202–1-AP, 1:1000), β-actin (Proteintech, 81115–1-RR, 1:5000), AMPK (Proteintech, 10929–2-AP, 1: 1000), GABARAP (Proteintech, 18723–1-AP, 1:500), Cleaved-Caspase3 (CST, 9664, 1:1000), Bcl-2 (Proteintech, 26593–1-AP, 1:1000), Bax (Proteintech, 50599–2-Ig, 1:1000), hVps41 (Proteintech, 13869–1-AP, 1:500), hVps11 (Proteintech, 19140–1-AP, 1:500), Rab7 (Proteintech, 55469–1-AP, 1:500), LC3 (Novus, NB100-2220, 1:1000) and p62 (CST, 88588, 1:1000).

### RT-PCR

RT-PCR were performed according to previous study (Ding et al. 2020).

### Transwell assay

Serum-free medium containing cells were added into the upper chamber (8 μm filter, Costar, Cambridge, MA, United States), followed by the addition of diluted Matrigel matrix gel (Corning, USA) in an incubator overnight. The medium containing 20% FBS was spread in the lower chamber. After the cell suspension was prepared, the cells were spread in the upper chamber. Non-migrating cells in the upper chamber were erased, and the migrated cells were fixed with 4% paraformaldehyde and stained with crystal violet (Procell, Wuhan, China).

### TUNEL assay

After washing the cells once with PBS (Procell, Wuhan, China), the cells were fixed with 4% paraformaldehyde (Beyotime, Shanghai, China) for 30 min and then washed once more with PBS. PBS with 0.1% Triton X-100 (Beyotime, Shanghai, China) was added and incubated for 2 min on ice. 50 μl of the ready-made TUNEL assay solution (Beyotime, Shanghai, China) was added and incubated at 37° C in the dark for 60 min before washing three times with PBS. TUNEL-positive cells were observed under fluorescence microscope after being treated with anti-fluorescence quenching solution.

### CCK-8

The protocol of CCK-8 was according to previous study (Ding et al. 2020).

### Immunofluorescence

Cells that had been seeded onto glass coverslips were fixed in 4% paraformaldehyde (Beyotime, Shanghai, China) for 10 min and then washed with PBS. The details referred to our previous study (Ding et al. 2020).

### LC3 track

GFP-RFP-LC3 lentivirus was purchased from Hanhang Biotech (Shanghai, China) and then transfected into cells and incubated at 37° C for 72 h. LSM-510 (Zeiss) confocal fluorescence microscope was used to observe the change of GFP-RFP-LC3 fusion protein in the autophagy stream. GFP (green light) was quenched in acidic environment, indicating that autophagy activity in cells was strong.

### Flow cytometry

Annexin V-FITC Apoptosis Detection Kit was purchased from Beyotime, Shanghai, China. The details referred to our previous study (Ding et al. 2020).

### ChIP assays

Chromatin Immunoprecipitation (ChIP) Assay Kit (Beyotime, Shanghai, China) was used to performe ChIP. The cultured cells were fixed with 37% formaldehyde (final concentration 1%) for 10 min, then the cross-linking reaction was terminated by adding 2 mol/L glycine solution (final concentration 0.125 mol/L), and the cells were incubated for 10 min. The solution was washed twice by adding PBS, centrifuged at 1000 × g for 20 min and the precipitate was subsequently resuspended in M2 buffer. This step was performed twice. DNA was interrupted with an ultrasonic crusher and diluted threefold with ChIP dilution buffer. 100 μL of Protein A/G Dynabeads was washed with 1 mL ChIP dilution buffer, and magnetic beads resuspended in 100 μL ChIP dilution buffer were added to prehybridize chromatin for 1 h. 20 μL of 5 mol/L NaCl was added, and the cross-linking was deactivated at 65 °C for 6 h. After decross-linking, the cells were placed in a -20 °C refrigerator for temporary storage. 20 mg/mL glycogen sedimentation aid was added, then 3 mol/L NaAc (pH5.2) was added at a 1:10 volume ratio, and finally 1 mL absolute ethanol was added, and precipitated at -20 °C for 3 h. The DNA was purified by washing twice with 70% ethanol. The degree of DNA enrichment was analyzed by quantitative PCR.

### CO-IP

After washing the cells twice with PBS, precooled RIPA (Beyotime, Shanghai, China) was added. Cells and suspensions were separated. Centrifugation was performed at 14000 rpm for 15 min at 4° C, and the supernatant was transferred to a new centrifuge tube. A 50% Protein A/G agarose bead working solution was added to the sample and the Protein A/G agarose beads were removed after centrifugation for 15 min. Antibodies were added and the mixture was incubated overnight. 100 μl Protein A agarose beads were added to capture the antibody-antibody complex, the antibody-antibody mixture was slowly shaken at 4° C for 1 h, and the precipitate was collected by transient centrifugation at 14000 rpm for 5 s, and the precipitate was washed three times with precooled wash buffer. The agarose beads-antigen–antibody complex was suspended in 60 μl of 2 × loading buffer and gently mixed. Free antigens, antibodies and beads were centrifuged, the supernatant was electrophorized, and the remaining agarose beads were collected. Later electrophoresis was carried out. Before electrophoresis, it should be boiled for 5 min again.

### Cholesterol analysis

Cholesterol was detected according to manufacturer’s protocol (Beyotime, Shanghai, China).

### Statistical analysis

SPSS 20.0 statistical software was used for statistical analysis, and all experiments were repeated at least three times. Data were expressed as mean ± standard deviation (mean ± SD). Analysis of variance and Tukey post hoc were used to analyze differences among multiple groups. When the *p*-value < 0.05. ***p < 0.001, **p < 0.01, *p < 0.05, the difference was considered statistically significant.

## Results

### The atherosclerosis dataset from GEO were analyzed

**Fig. 1:**
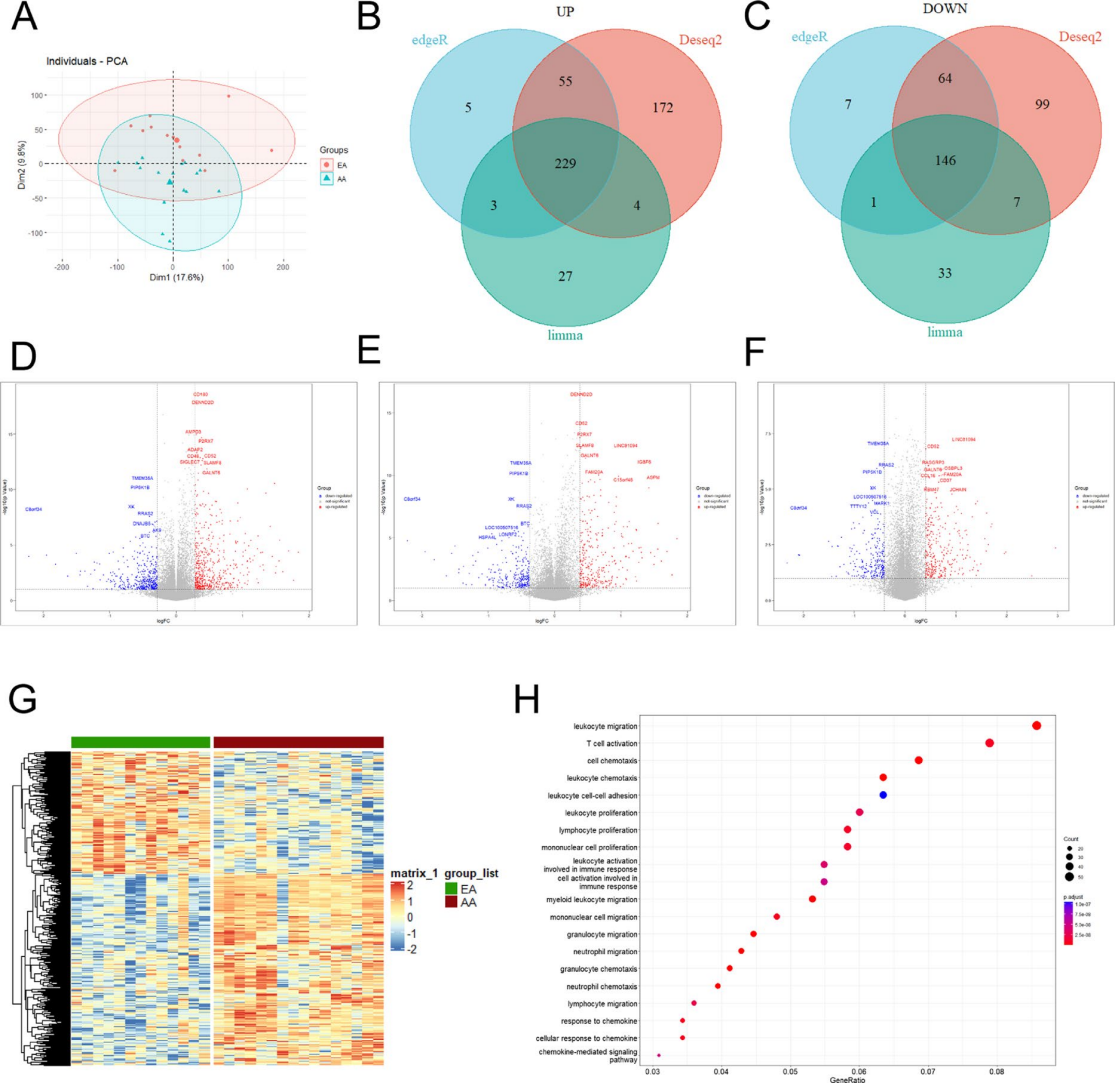
The results of analysis to GSE28829 (**A**) Feature distribution profile of early atherosclerosis genomes and advanced atherosclerosis genomes (PERMANOVA *p*-value < 2.8e^−13^ (**B**-**C**) DESeq2, edgeR, and Limma R packages were used to analyze GSE28829 datasets. The Venn diagram of highly expressed genes and lowly expressed genes were shown. (**D**-**F**) The differentially expressed analysis results of GSE28829 by DESeq2, edgeR, and Limma R packages were shown by volcanic plot. |log FC (a fold change) |= 1.0 and *P* < 0. 05. (**G**) The results of differentially expressed genes were shown by heatmap. (*H*) GO enrichment analysis of differentially expressed genes

In order to obtain relevant data in atherosclerotic tissues and normal tissues, data sets numbered were downloaded from the GEO database. PCA plots of the sample characteristics for the three microarrays showed that three microarrays were clearly grouped (Fig. 1A, Fig. S1A and S2A). To reduce analysis error, edgeR, DESeq2, and limma was used and the results showed that the GSE28829 dataset had 229 highly expressed genes (Fig. 1B) and 146 lowly expressed genes (Fig. 1C). The GSE43292 dataset had 359 highly expressed genes (Fig. S1B) and 636 lowly expressed genes (Fig. S1C). The GSE41571 dataset had 403 highly expressed genes (Fig. S2B) and 378 lowly expressed genes (Fig. S2C). | log FC (a fold change) |= 1.0 and P value < 0. 05 were set. Three datasets including total of 2151 differentially expressed genes were analyzed and was shown by heatmaps (Fig. 1G, Fig. S1G and S2G). The differentially expressed analysis was performed on the three microarray datasets and the corresponding volcano plot was obtained (Fig. 1D-F, Fig. S1D-F and S2D-F). Subsequently, KEGG analysis of differentially expressed genes was performed, and no abnormal cell signaling were found (Fig. 1H, Fig. S1H and S2H).

### MYBL1 were downregulated in the plaque of atherosclerosis

**Fig. 2:**
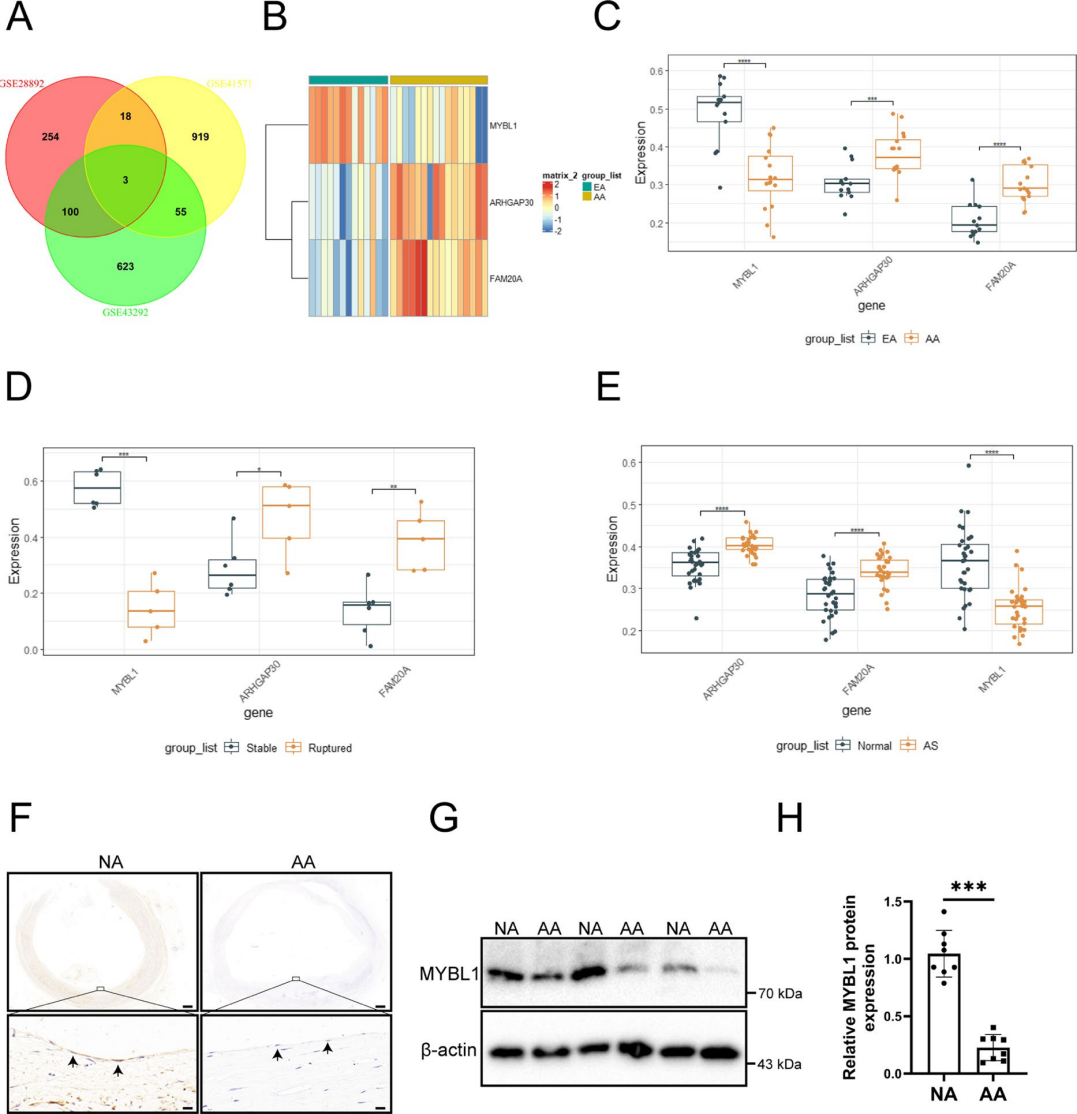
Different expression of MYBL1, ARHGAP30 and FAM20A was observed in GSE28829, GSE43292 and GSE41571 datasets (**A**) The intersection of differentially expressed genes of GSE28829, GSE43292 and GSE41571 datasets was selected. (**B**) Heat map of MYBL1, ARHGAP30 and FAM20A genes in early and late atherosclerotic tissues. (**C**-**E**) GEPIA analysis of the three genes MYBL1, ARHGAP30, and FAM20A was performed to obtain box plots of their expression. (**F**) Immumohistochemical staining was used to determine the protein expression of MYBL1 in advanced atherosclerotic plaques (AA) and normal atherosclerotic plaques (NA) (*n* = 8). Scale bars = 200 μm or 20 μm. (**G**)The protein expression of MYBL1 was detected by western blot in early atherosclerosis and advanced atherosclerosis (*n* = 8). (**H**) The quantitative analysis of MYBL1 protein expression in early atherosclerosis and advanced atherosclerosis were shown. *p < 0.05, **p < 0.01, ***p < 0.001, ****p < 0.0001

The differentially expressed genes of the three datasets were intersected, and three genes were obtained (Fig. 2A), which were MYBL1, ARHGAP30, and FAM20A. which of the three genes have an effect on atherosclerosis remained unclear and three genes into samples for validation should be confirmed. Firstly, heatmap analysis of the three differential genes including MYBL1, ARHGAP30 and FAM20A were performed and found that MYBL1 had the highest expression in early atherosclerotic tissues and the lowest expression in advanced atherosclerosis, while FAM20A and ARHGAP30 had the opposite trend (Fig. 2B). GEPIA analysis was performed to obtain the box plot of their expression. MYBL1 expression was significantly decreased in early atherosclerotic tissues. MYBL1 expression was significantly reduced in advanced atherosclerotic tissues. MYBL1 showed a more significant difference than ARHGAP30 and FAM20A (Fig. 2C). The expression of MYBL1 was significantly higher in stable atherosclerotic plaque tissue. In ruptured atherosclerotic plaque tissue, MYBL1 also showed a more significant difference than ARHGAP30 and FAM20A (Fig. 2D). What’s more, in normal HUVECs, MYBL1 showed a more significant difference than ARHGAP30 and FAM20A in atherosclerotic plaques (Fig. 2E). These results suggests that the downregulation of MYBL1 expression was most pronounced in atherosclerotic plaques. Subsequently, Western blot was used to verify MYBL1 protein expression, and the results showed that MYBL1 protein expression was weaker in advanced atherosclerotic plaques (AA) than in normal atherosclerotic plaques (NA) (Fig. 2F, G and H). These results indicated that MYBL1 expression was decreased in atherosclerotic plaque tissue.

### Silence of MYBL1 caused damage to endothelial cells

**Fig. 3:**
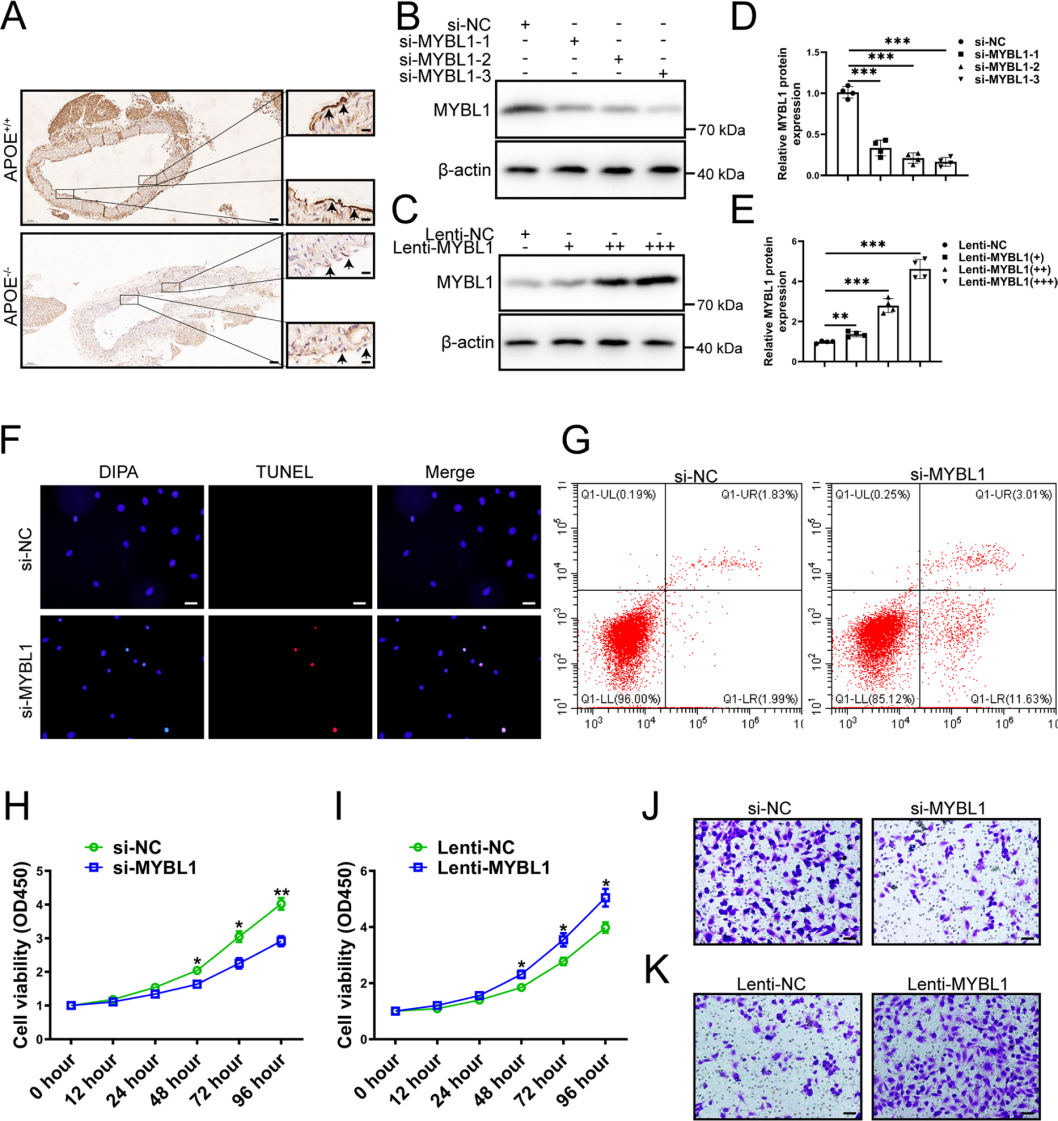
MYBL1 was involved in the development of atherosclerosis (**A**) Immunohistochemistry was performed on APOE^+/+^ and APOE.^−/−^ to assess MYBL1 protein expression in atherosclerotic plaques (*n* = 7). Scale bars = 200 μm or 20 μm. (**B**) The effect of si-MYBL1-1, si-MYBL1-2, and si-MYBL1-3 were confirmed by western blot (*n* = 4). (**C**) The effect of Lenti-MYBL1 were confirmed by western blot (*n* = 4). (D-E) The quantitative analysis of MYBL1 protein expression were shown. (**F**) TUNEL assay of HUVECs showed that MYBL1 inhibition induced apoptosis (*n* = 5). Scale bars = 20 μm. (**G**) Flow cytometry was used to detect the apoptosis rate of HUVECs when MYBL1 was inhibited, and the results showed that the apoptosis rate of HUVECs was higher after MYBL1 inhibition (*n* = 5). (H-I) CCK-8 was used to detect the viability of HUVECs after transfection of si-MYBL1 or Lenti-MYBL1 (*n* = 3). (**J**-**K**) Transwell migration assay was used to detect HUVECs migration ability after transfection of si-MYBL1 or Lenti-MYBL1 (*n* = 5). Scale bars = 100 μm. *p < 0.05, **p < 0.01, ***p < 0.001

The mechanism of how MYBL1 downregulation leads to atherosclerosis remains unknown. To determine the relationship between MYBL1 and vascular endothelial cells, MYBL1 were examined by immunohistochemistry. As shown in Fig. 3A, MYBL1 content was significantly lower in advanced atherosclerotic plaques of APOE^−/−^ mice than in controls. In order to verify the function of MYBL1, small interfering RNA (si-RNA) and lentivirus-overexpressed system was synthesized, and found that si-MYBL1s could inhibit the expression of MYBL1 in HUVECs, among which si-MYBL1-3 had the most obvious effect (Fig. 3B and D). What’s more, Lenti-MYBL1 could promote the expression of MYBL1 with the increase of Lenti-MYBL1 concentration in HUVECs (Fig. 3C and E). Si-MYBL1-3 and high concentration of Lenti-MYBL1 was used for subsequent experiments.

MYBL1 was subsequently silenced and overexpressed to evaluate the endothelial cells response. TUNEL assay showed that MYBL1 inhibition induced apoptosis (Fig. 3F). The results of Fig. 3G showed that the apoptosis rate of HUVECs was higher after MYBL1 inhibition. The results showed that after MYBL1 inhibition, the activity of HUVECs was weakened, and the proliferation ability was weaker than that of the control cells (Fig. 3H). After infection with Lenti-MYBL1, the activity of HUVECs was enhanced, and their migration ability was stronger than that of the control cells (Fig. 3I). Transwell migration assay verified the following results: after silencing of MYBL1 for 72 h, the migration ability of HUVECs was weakened (Fig. 3J), while MYBL1 overexpression caused the migration ability of HUVECs to be stronger (Fig. 3K). Zhu et al. also demonstrated that silencing of MYBL1 resulted in the reduced migration ability of HUVECs (Zhu et al. 2022). These results suggested that silencing of MYBL1 could weaken the proliferation and migration of HUVECs, and even lead to apoptosis and damage HUVECs. However, further experiments were needed to understand how MYBL1 causes endothelial cell damage.

### MYBL1 might involve in the regulation of endothelial cell by autophagy

**Fig. 4:**
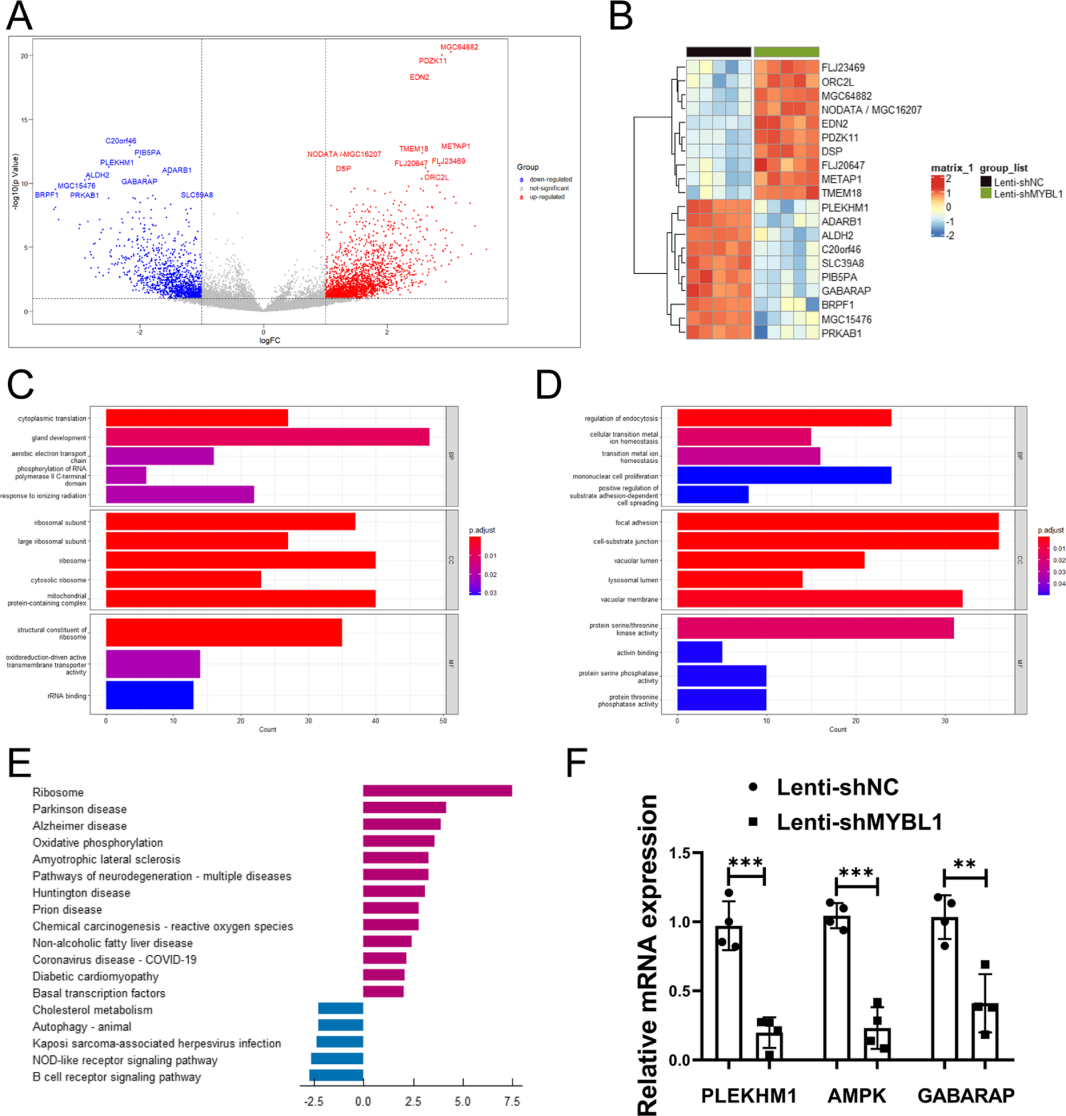
MYBL1 might regulate in the development of atherosclerosis by autophagy (**A**) Volcano map to differentially expressed genes of transcriptome sequencing after MYBL1 knockdown was shown (n = 5). (**B**) Heatmap to top 10 up-regulated and top 10 down-regulated genes of transcriptome sequencing after MYBL1 knockdown was shown. (**C**) GO enrichment analysis to up-regulated genes were shown. (**D**) GO enrichment analysis to down-regulated genes were shown. (**E**) KEGG enrichment analysis to differentially expressed genes were shown. (**F**) PLEKHM1, AMPK and GABARAP RNA expression in HUVECs transfected with Lenti-shMYBL1 or Lenti-shNC. *p < 0.05, **p < 0.01, ***p < 0.001

Next, the HUVECs after MYBL1 knockdown were subjected to transcriptome sequencing, and the corresponding volcano map of differentially expressed genes was obtained (Fig. 4A). Subsequently, top 10 genes were selected with high and low expression and obtained a heat map of these 20 genes (Fig. 4B). Then, GO analysis (Fig. 4C-D) was performed on the high-expression genes and low-expression genes detected by transcriptome sequencing. Then KEGG enrichment analysis of genes were performed and found that autophagy-related processes were inhibited after MYBL1 knockdown (Fig. 4E), among which PLEKHM1, AMPK and GABARAP were the three most significant genes affecting the changes of autophagy pathway (Fig. 4B). Then the three genes were validated to be expressed in HUVECs and three lower mRNA expression of PLEKHM1, AMPK and GABARAP was confirmed. It was found that after MYBL1 knockdown, the mRNA expression of PLEKHM1, AMPK and GABARAP was down-regulated compared with the control group, and the expression of PLEKHM1 was lower than that of AMPK and GABARAP both in the control group and after MYBL1 knockdown (Fig. 4F). Therefore, we proposed that MYBL1 might be involved in the regulation of endothelial cells through autophagy.

### PLEKHM1 served as a downstream of MYBL1 in endothelial cells

**Fig. 5:**
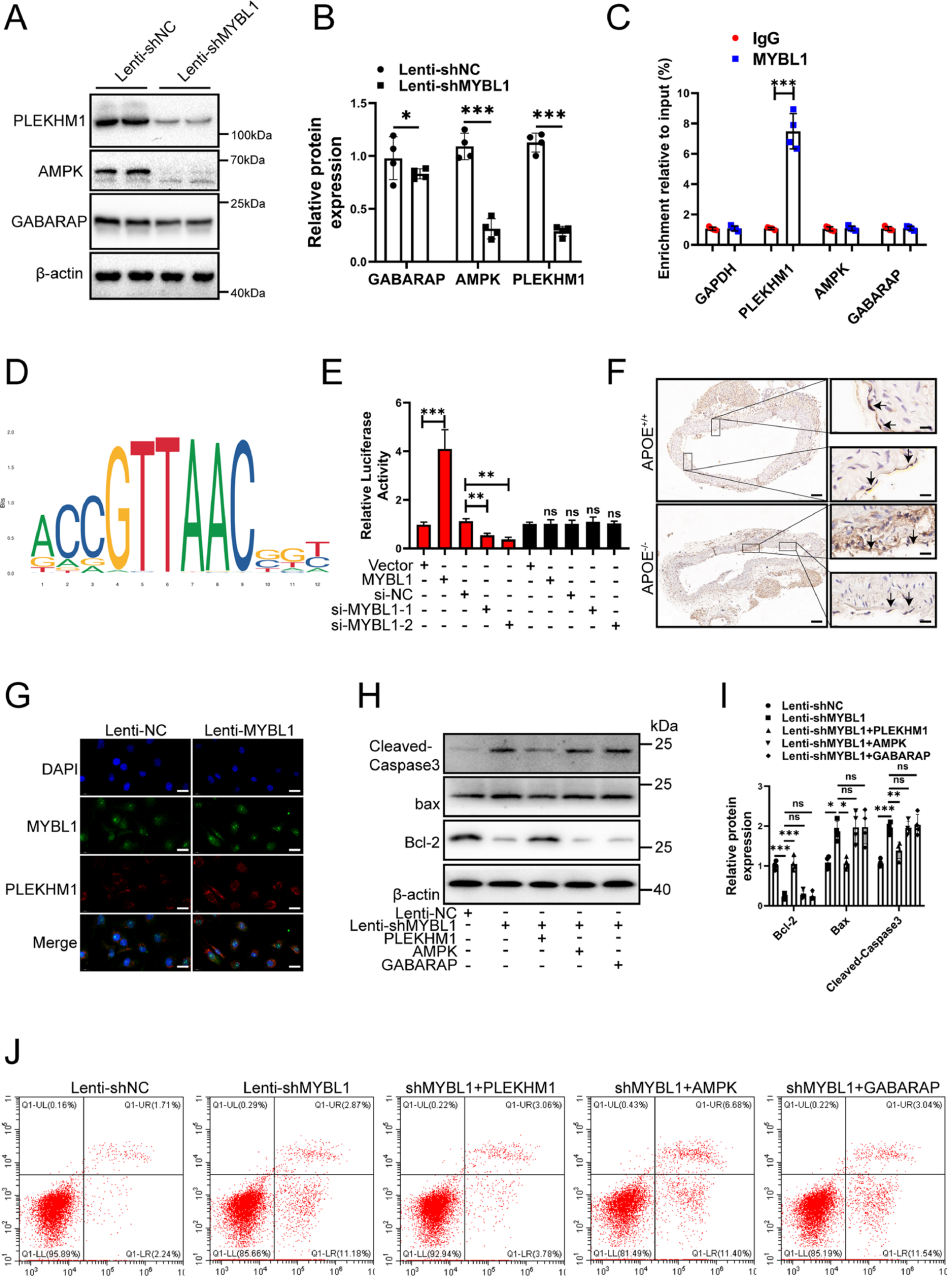
PLEKHM1 might act as a target of MYBL1 to regulate MYBL1 (**A**) Western blot detected the expressions of PLEKHM1, AMPK, GABARAP and β-actin when MYBL1 was overexpressed and the negative control (*n* = 4). (**B**) The quantitative analysis of PLEKHM1, AMPK and GABARAP protein expression were shown. (**C**) CHIP assay was used to detect the interaction between MYBL1 and the promoters of GAPDH, PLEKHM1, AMPK and GABARAP genes (*n* = 3). (**D**) PLEKHM1 corresponds to DNA binding site information map. (**E**) Luciferase reporter system was performed to examine the combination between PLEKHM1 and MYBL1 (*n* = 3). (**F**) Immunohistochemistry was performed on APOE^+/+^ and APOE.^−/−^ to assess PLEKHM1 protein expression in atherosclerotic plaques (*n* = 8). Scale bars = 200 μm or 20 μm. (**G**) Immunofluorescence assay was used to detect whether MYBL1 entered the nucleus (*n* = 3). Scale bars = 200 μm or 20 μm. (**H**) Western blot was used to detect the apoptosis of cells with PLEKHM1, AMPK and GABARAP overexpression when MYBL1 was inhibited (*n* = 4). (*I*) The quantitative analysis of Cleaved-Caspase3, Bax and Bcl2 protein expression were shown. (**J**) Flow cytometry was used to detect the apoptosis rate of HUVECs (*n* = 5). *p < 0.05, **p < 0.01, ***p < 0.001

Next, which of the PLEKHM1, AMPK and GABARAP genes was involved in the regulation of endothelial cells as the target gene of MYBL1 were verified. Western blot was performed to detect the protein expression of PLEKHM1, AMPK and GABARAP after MYBL1 knockdown. The results showed that after MYBL1 knockdown, the protein expressions of PLEKHM1, AMPK and GABARAP were decreased, and the expressions of PLEKHM1 and AMPK were more significantly decreased (Fig. 5A and B). In order to further clarify which gene promoters MYBL1 binded to, CHIP experiments were performed. The results showed that the expression of PLEKHM1 was the most significant and MYBL1 interacted with the promoter on PLEKHM1 (Fig. 5C). PLEKHM1 corresponds to DNA binding site information map was shown (Fig. 5D). The results of luciferase reporting system showed that upregulation of MYBL1 increased obviously the luminescence of luciferase. The luminescence of luciferase was significantly inhibited after MYBL1 inhibition. In the negative control group, the luminescence of luciferase was not obvious (Fig. 5E). This means that MYBL1 bound to the PLEKHM1 promoter. PLEKHM1 protein expression was obviously lower in APOE^−/−^ and APOE^+/+^mice (Fig. 5F). The results of immunofluorescence showed that MYBL1 continued to glow after MYBL1 was upregulated, and the brightness was stronger than that of the control group, indicating that MYBL1 were in the nucleus (Fig. 5G). Then western blot was conducted and the results revealed that overexpression of AMPK and GABARAP could not reduce apoptosis after MYBL1 knockdown. However, PLEKHM1 overexpression inhibited apoptosis after MYBL1 knockdown (Fig. 5H and I). The results of flow cytometry showed that the apoptosis induced by MYBL1 was reduced after upregulation of PLEKHM1 but upregulation of AMPK or GABARAP could not reduce (Fig. 5J). These results revealed that PLEKHM1 acted as a target of MYBL1 to protect HUVECs.

### PLEKHM1 promoted the fusion of autophagy and lysosomes in endothelial cells

**Fig. 6:**
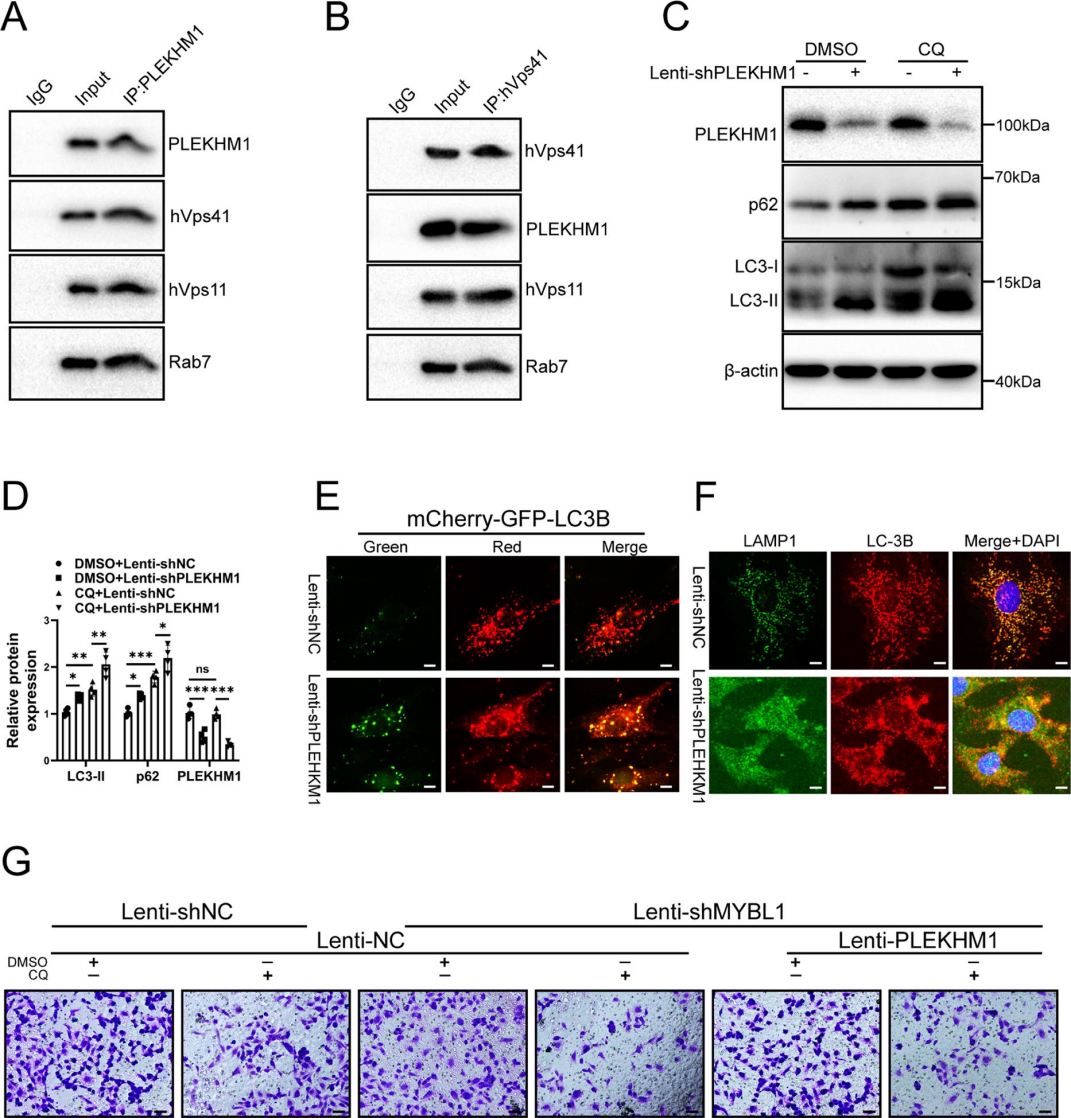
MYBL1 upregulated the function of HUVECs through PLEKHM1-induced autophagy (**A**) CO-IP was used to detect the binding of PLEKHM1 to hVps41, hVps11 and Rab7 (*n* = 3). (**B**) CO-IP was used to detect the binding of hVps41 to PLEKHM1, hVps11 and Rab7 (*n* = 3). (**C**) Western blot was used to detect the protein expression of PLEKHM1, p62, LC-3 and β-actin in HUVECs after treatments (*n* = 4). (**D**) The quantitative analysis of PLEKHM1, p62 and LC-3II were shown. (**E**) Track to autophagy performed by mCherry-GFP-LC3B was shown (*n* = 3). Scale bars = 10 μm. (**F**) The location and expression of LAMP1 and LC3B was determined by immunofluorescence (*n* = 3). Scale bars = 10 μm. (**G**) The migration of HUVECs was determined by Transwell assay after treatments (*n* = 5). *p < 0.05, **p < 0.01, ***p < 0.001

Although previous studies showed that PLEKHM1 was one of the genes with the most significant changes in the autophagy pathway (McEwan et al. 2014; Fas et al. 2020; Ho et al. 2020), how PLEKHM1 regulates autophagy was still unclear, so more in-depth experiments were needed. Co-IP experiments showed that PLEKHM1 and hVps41 were interacting proteins that promoted autophagosome and lysosomal fusion in HUVECs (Fig. 6A and B). Inhibition of autophagy by Chloroquine (CQ, 10 µM) in HUVECs was according to previous study (He et al. 2019). Then PLEKHM1 was knocked down, and added DMSO or CQ in HUVECs for western blot. The results showed that CQ inhibited the downstream of autophagic flow and led to the increase of p62 and LC3-II expression (Fig. 6C and D). Detection of autophagic flux in HUVECs using lentivirus carrying mCherry-GFP-LC3B showed decreased autophagic flux after knockdown of PLEKHM1 (Fig. 6E). The results of immunofluorescence showed that the positions of autophagy marker protein LC3 and lysosomal marker protein LAMP1 were highly coincident (Fig. 6F), suggesting that autophagy and lysosomes were fused. Transwell assay showed that MYBL1 knockdown reduced the migration ability of HUVEC cells. Knockdown of both MYBL1 and PLEKHM1 further reduced cell migration (Fig. 6G). These results suggest that PLEKHM1, a target gene of MYBL1, protected HUVECs by promoting autophagosome and lysosomal fusion.

### MYBL1/PLEKHM1 signal pathway reduced lipid accumulation in HUVECs

**Fig. 7:**
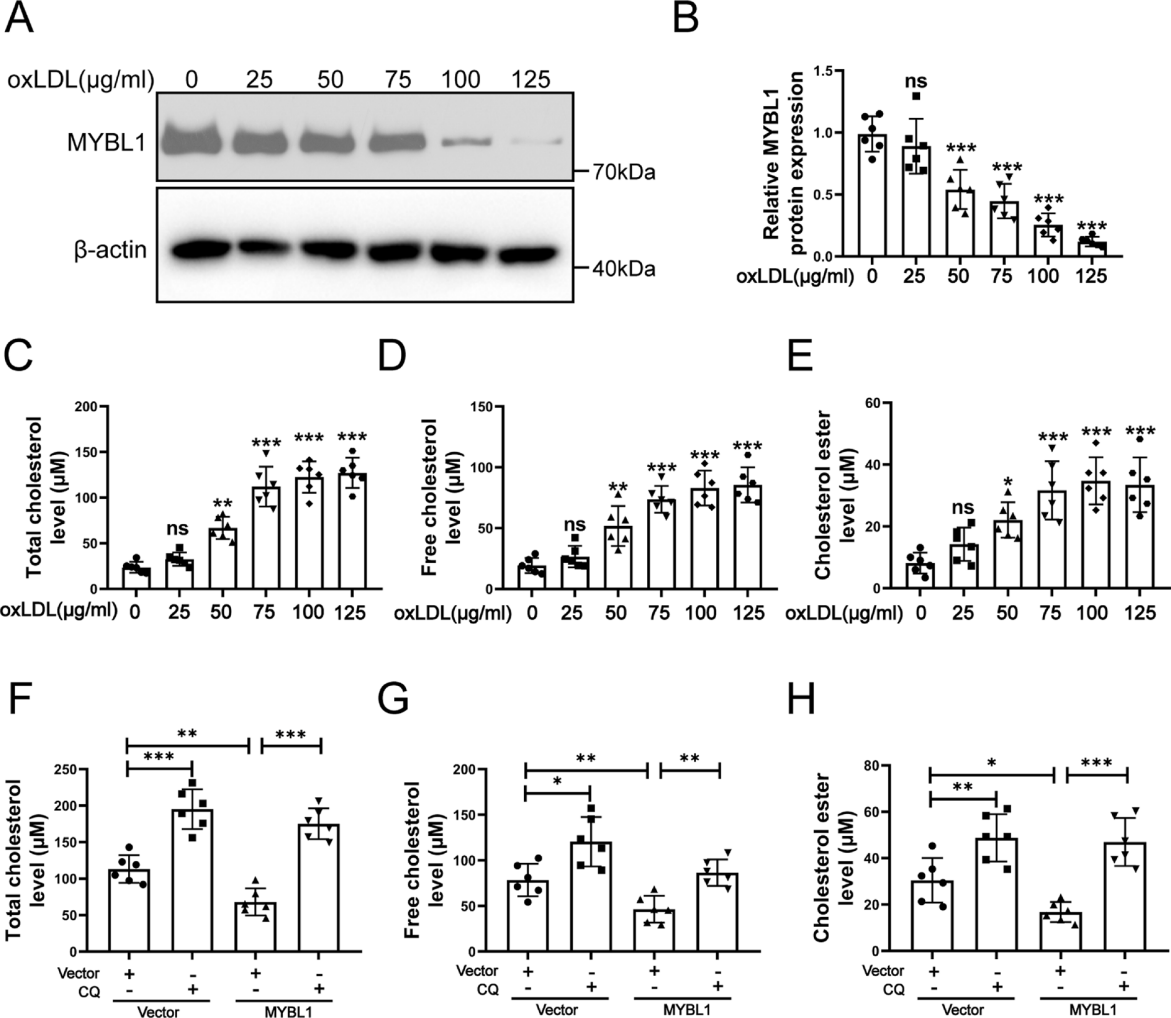
MYBL1 might regulated lipid accumulation by autophagy in HUVECs after oxLDL treatment (**A**) MYBL1 protein expression were determined by Western blot in HUVECs treated with oxLDL (0, 25, 50, 75, 100 and 125 μg/ml) (*n* = 6). (**B**) The quantitative analysis of MYBL1 protein expression were shown. (**C**-**E**) The total cholesterol, free cholesterol and cholesterol ester levels in HUVECs treated with oxLDL were determined by HPLC (*n* = 6). (F–H) The total cholesterol, free cholesterol and cholesterol ester levels in HUVECs were determined by HPLC. HUVECs were transfected with Lenti-NC or Lenti-MYBL1.Then HUVECs with vector or CQ. HUVECs were finally treated by oxLDL (100 μg/ml) (*n* = 6). *p < 0.05, **p < 0.01, ***p < 0.001

Lipid accumulation destroy the physiological function of HUVECs and increase the apoptosis of HUVECs in the atherosclerosis (Zhang et al. 2023; Yu et al. 1993; Chiu et al. 2023). KEGG enrichment analysis of GSE28829, GSE43292 and GSE41571 showed that lipid metabolism was involved in atherosclerosis (Fig. S3 and Fig. S4). To verify whether MYBL1 protected HUVECs by regulating lipid metabolism, HUVECs were treated with oxLDL (oxidized low-density lipoprotein) at different concentrations (0, 25, 50, 75, 100 and 125 μg/ml). The protein expression of MYBL1 was determined by Western blot. MYBL1 protein expression was gradually decreased in HUVECs treated with oxLDL at different concentrations (Fig. 7A and B). OxLDL also caused a gradual increase of total cholesterol (TC), free cholesterol (FC) and cholesterol ester (CE) levels of HUVECs (Fig. 7C-E). Because lipophagy is the main mechanism of LD degradation, and most cancer cells exhibit higher levels of basal autophagy than normal cells (Singh et al. 2009; White 2012). Combined with above results, we speculated that MYBL1 reduced lipid accumulation through lipophagy. CQ could increase the levels ofTC, FC and CE although upregulation of MYBL1 in HUVECs (Fig. 7F-G).

**Fig. 8:**
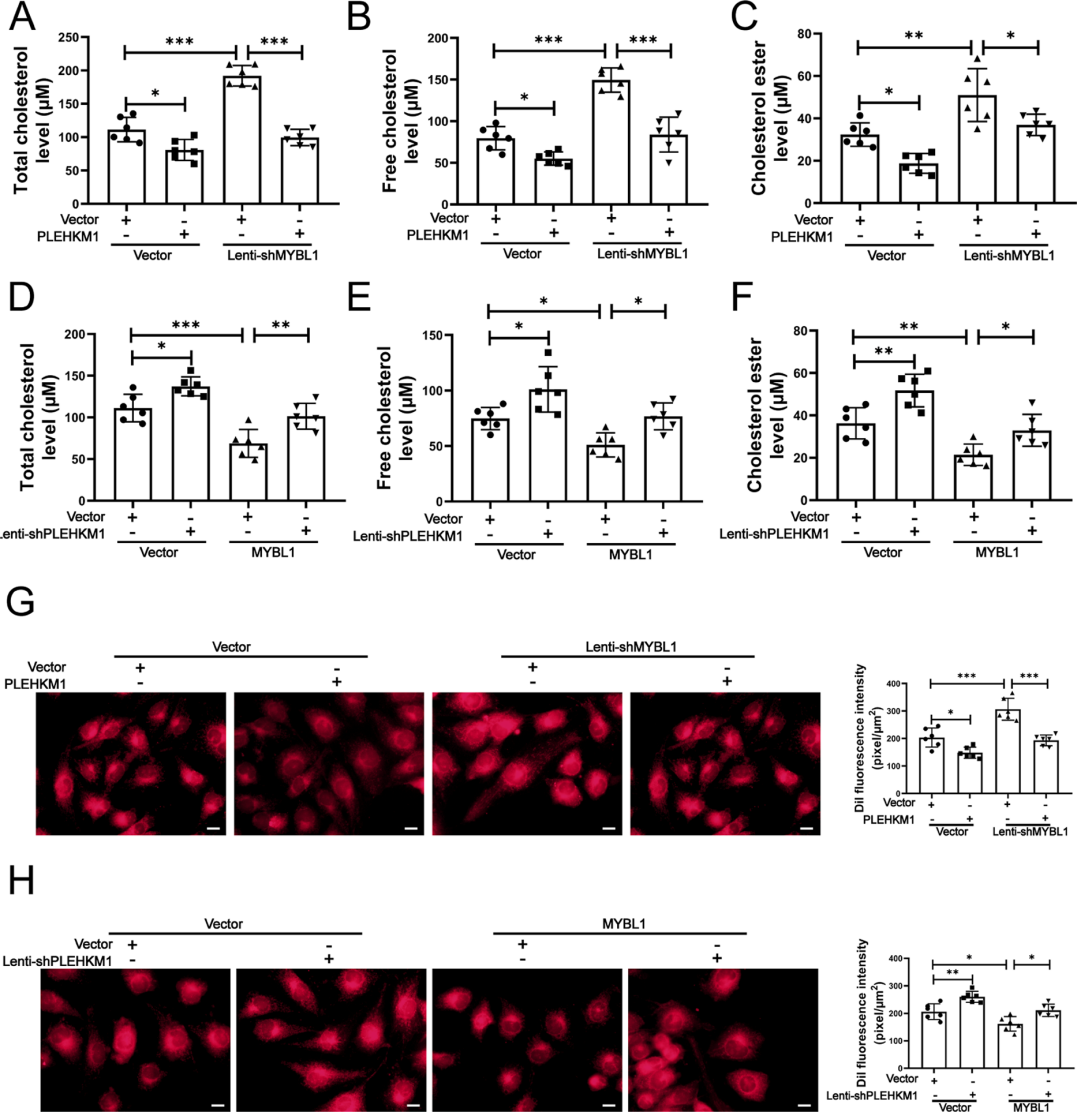
MYBL1/PLEKHM1 signal pathway reduced lipid accumulation in HUVECs (A-C) The total cholesterol, free cholesterol and cholesterol ester levels in HUVECs were determined by HPLC. HUVECs were transfected with Lenti-shNC or Lenti-shMYBL1.Then HUVECs with Lenti-shNC or Lenti-shMYBL1 were transfected with Lenti-NC or Lenti-PLEHKM1. HUVECs were finally treated by oxLDL (100 μg/ml) (*n* = 6). (D-F) The total cholesterol, free cholesterol and cholesterol ester levels in HUVECs were determined by HPLC. HUVECs were transfected with Lenti-NC or Lenti-MYBL1.Then HUVECs with Lenti-NC or Lenti-MYBL1 were transfected with Lenti-shNC or Lenti-shPLEHKM1. HUVECs were finally treated by oxLDL (100 μg/ml) (*n* = 6). (G) DiI-ox-LDL staining in HUVECs was evaluated. The DiI fluorescence intensity was quantified with Image Pro Plus. HUVECs were transfected with Lenti-shNC or Lenti-shMYBL1.Then HUVECs with Lenti-shNC or Lenti-shMYBL1 were transfected with Lenti-NC or Lenti-PLEHKM1. HUVECs were finally treated by oxLDL (100 μg/ml) (*n* = 6). (H) DiI-ox-LDL staining in HUVECs was evaluated. The DiI fluorescence intensity was quantified with Image Pro Plus. HUVECs were transfected with Lenti-NC or Lenti-MYBL1.Then HUVECs with Lenti-NC or Lenti-MYBL1 were transfected with Lenti-shNC or Lenti-shPLEHKM1. HUVECs were finally treated by oxLDL (100 μg/ml) (*n* = 5). Scale bars = 20 μm. *p < 0.05, **p < 0.01, ***p < 0.001

Silencing of MYBL1 increased the levels of TC, FC and CE while upregulation of PLEKHM1 reduced the levels of TC, FC and CE in HUVECs transfected with Lenti-shMYBL1 (Fig. 8A, B and C). In addition, upregulation of MYBL1 reduced the levels of TC, FC and CE and silencing of PLEKHM1 increased the levels of TC, FC and CE in HUVECs transfected with Lenti-MYBL1 (Fig. 8D, E and F). Furthermore, we found that silencing of MYBL1 increased the DiI-derived fluorescence intensity and upregulation of PLEKHM1 reduced the DiI-derived fluorescence intensity in HUVECs transfected with Lenti-shMYBL1 (Fig. 8G). Upregulation of MYBL1 reduced the DiI-derived fluorescence intensity and silencing of PLEKHM1 increased the DiI-derived fluorescence intensity in HUVECs transfected with Lenti-MYBL1 (Fig. 8H). These results showed that MYBL1/PLEKHM1 signal pathway reduced lipid accumulation in HUVECs.

## Discussion

Atherosclerosis is an inflammatory disease that occurs in the lining of blood vessels, usually resulting in the formation of plaques rich in cells and lipids (Libby et al. 2002; Li et al. 2022). For a long time, many researchers have devoted themselves to the field of atherosclerosis in order to elucidate the mechanism of atherosclerosis. Doring et al. processed 17 atherosclerotic tissue samples including 9 early atherosclerosis and 8 advanced atherosclerosis to obtain GSE28829 (Döring et al. 2012). AYARI et al. divided 34 carotid endarterectomy specimens from Hospital Edouard Herriot patients into atherosclerotic plaque tissue and normal tissue, and serially analyzed the tissue to obtain GSE43292 (Ayari and Bricca 2013). K. Lee et al. generated GSE41571 (Lee et al. 2012) from stable and ruptured atherosclerotic plaques. It can be seen that although the three datasets are all studies on atherosclerosis, the sample characteristics are different and each is not representative enough. Therefore, in order to make the sample data of this paper more extensive and comprehensive, the intersection of GSE28829, GSE43292 and GSE41571 datasets were took as the basis to obtain the differentially expressed genes. MYBL1, ARHGAP30 and FAM20A were selected from GSE28829, GSE43292 and GSE41571. GEPIA analysis of the three genes revealed that MYBL1 was down-regulated in atherosclerotic plaques and played an important role in HUVECs. The phenotype of HUVECs were impacted by knockdown and overexpression of MYBL1. MYBL1 knockdown inhibited HUVECs migration, activity and promoted apoptosis. In contrast, overexpression of MYBL1 by lentivirus promoted HUVECs migration and enhanced cell viability.

Subsequent next generation sequencing and functional enrichment analysis of HUVECs with MYBL1 knockdown showed that autophagy-related processes were inhibited, which led us to hypothesize that MYBL1 might protect HUVECs by inducing autophagy. Previous studies have shown that dysfunction of autophagy promotes atherosclerosis (Qiao et al. 2021), inhibits inflammasome-dependent inflammation (Razani et al. 2012) and promotes cholesterol efflux (Ouimet et al. 2011), thereby achieving anti-atherosclerotic effects. This is consistent with the results of our study. Among the differentially expressed genes, PLEKHM1, AMPK and GABARAP had the greatest influence on autophagy. Although all three genes promote autophagy, they act in different ways. AMP-activated protein kinase (AMPK) is the main sensor of cellular energy, which has evolved and been maintained in all eukaryotes through adenine nucleotide levels. Although AMPK regulates autophagy and mitochondrial homeostasis (Herzig and Shaw 2017), and can reshape cellular metabolism in a prolonged manner by targeting transcriptional regulators, AMPK works by phosphorylating substrates at specific key points (Jäger et al. 2007; Yang et al. 2001; Koo et al. 2005; Greer et al. 2007; Lamia et al. 2009; Bungard et al. 2010; Li et al. 2011; Mihaylova et al. 2011; Shin et al. 2016; Young et al. 2016). Activated AMPK regulates the activity of transcription factors. AMPK is upstream of the transcription factors. It has been shown that GABARAPs are the major drivers of autophagy type, and they interact with LC3 to recruit PLEKHM1 to autophagosomes (Trefts and Shaw 2021). PLEKHM1 then associates with homotypic fusion and protein sorting (HOPS) complex to promote autophagosome-lysosome fusion (Nguyen et al. 2016). PLEKHM1 regulates autophagosome-lysosome fusion during starvation autophagy, mitophagy and aggregation autophagy (McEwan et al. 2014). Herein, PLEKHM1 was confirmed to be a downstream gene of MYBL1 in endothelial cells by western blot, CHIP assay, immunofluorescence assay, and luciferase reporter assay. MYBL1 enhanced the binding of autophagosomes to lysosomes by PLEKHM1.

This study had two limitations. Firstly, atherosclerosis involved a variety of cells, three of which were macrophages, vascular smooth muscle cells and endothelial cells. In this study, the function of MYBL1 was only examined in HUVECs, but not in macrophages and VSMCS. Second, validation of MYBL1 in vivo was not performed in this study.

**Fig. 9:**
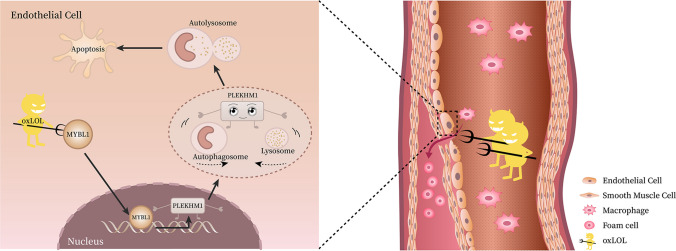
A schematic diagram for the underlying mechanism of the UPR^mt^ in regulating IVDD. OxLDL attacked MYBL1 to induce apoptosis of endothelial cells in the atherosclerosis. PLEKHM1, as a downstream of MYBL1 in endothelial cells, promoted the fusion of autophagy and lysosomes in endothelial cells

Overall, our study demonstrated that MYBL1 was inhibited in the vascular hyperlipemia environment, so that it could not enter the nucleus to bind PLEKHM1 promoter, which leaded to decreased PLEKHM1 expression, inhibition of lipid phagocytosis by autophagy, and finally induction of apoptosis in endothelial cells. Upregulation of MYBL1 might attenuate atherosclerotic plaque formation through PLEKHM1-induced autophagy (Fig. 9).

## Supplementary Information

Below is the link to the electronic supplementary material.

Supplementary file1 (TIF 1024 KB) The results of analysis to GSE43292 (A) Feature distribution profile of early atherosclerosis genomes and advanced atherosclerosis genomes (PERMANOVA p-value < 2.8e^-13^ (B-C) DESeq2, edgeR, and Limma R packages were used to analyze GSE43292 datasets. The Venn diagram of highly expressed genes and lowly expressed genes were shown. (D-F) The differentially expressed analysis results of GSE43292 by DESeq2, edgeR, and Limma R packages were shown by volcanic plot. |log FC (a fold change) | = 1.0 and P < 0. 05. (G) The results of differentially expressed genes were shown by heatmap. (H) GO enrichment analysis of differentially expressed genes

Supplementary file2 (TIF 1493 KB) The results of analysis to GSE41571 (A) Feature distribution profile of early atherosclerosis genomes and advanced atherosclerosis genomes (PERMANOVA p-value < 2.8e^-13^ (B-C) DESeq2, edgeR, and Limma R packages were used to analyze GSE41571 datasets. The Venn diagram of highly expressed genes and lowly expressed genes were shown. (D-F) The differentially expressed analysis results of GSE41571 by DESeq2, edgeR, and Limma R packages were shown by volcanic plot. | log FC (a fold change) | = 1.0 and P < 0. 05. (G) The results of differentially expressed genes were shown by heatmap. (H) GO enrichment analysis of differentially expressed genes

Supplementary file3 (TIF 555 KB) The results of KEGG enrichment analysis of differentially expressed genes (A) KEGG enrichment analysis of differentially expressed genes in GSE28829. (B) KEGG enrichment analysis of differentially expressed genes in GSE43292. (C) KEGG enrichment analysis of differentially expressed genes in GSE41571

Supplementary file4 (TIF 853 KB) Intersection of data set enrichment analysis and related enrichment pathways (A) The intersection of GO enrichment analysis between GSE28829, GSE43292 and GSE41571 datasets was selected. (B) The intersection of KEGG enrichment analysis to highly expressed genes between GSE28829, GSE43292 and GSE41571 datasets was selected. (C) The intersection of KEGG enrichment analysis to lowly expressed genes between GSE28829, GSE43292 and GSE41571 datasets was selected. (D) 116 signal pathway of GO enrichment analysis was shown. (E) 23 intersected KEGG enrichment analysis was shown

## Data Availability

The Microarray data are publicly accessible via the Gene Expression Omnibus, no. GSE28829, GSE43292 and GSE41571. The datasets generated during and/or analyzed during the current study are available from the corresponding author on reasonable request.
