# Supplementary figures and images for: Downregulation of MYBL1 in endothelial cells contributes to atherosclerosis by repressing PLEKHM1-inducing autophagy
Source: Cell Biol Toxicol. 2024 May 27;40(1):40. doi: 10.1007/s10565-024-09873-6 (PMC11128406; doi:10.1007/s10565-024-09873-6)

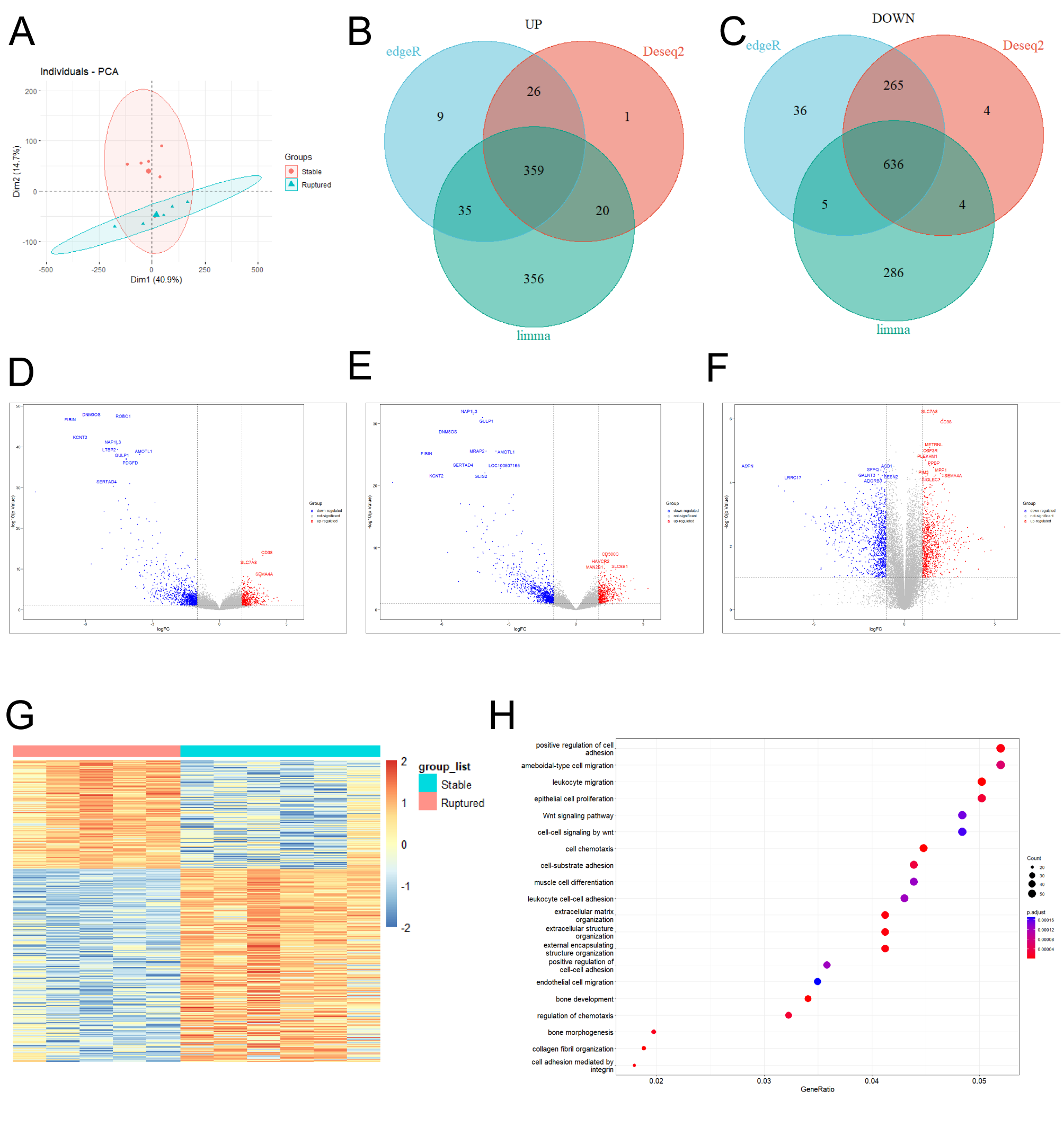

Supplement: Supplementary file 1 — Supplementary file1 (TIF 1024 KB) The results of analysis to GSE43292 (A) Feature distribution profile of early atherosclerosis genomes and advanced atherosclerosis genomes (PERMANOVA p-value < 2.8e-13 (B-C) DESeq2, edgeR, and Limma R packages were used to analyze GSE43292 datasets. The Venn diagram of highly expressed genes and lowly expressed genes were shown. (D-F) The differentially expressed analysis results of GSE43292 by DESeq2, edgeR, and Limma R packages were shown by volcanic plot. |log FC (a fold change) | = 1.0 and P < 0. 05. (G) The results of differentially expressed genes were shown by heatmap. (H) GO enrichment analysis of differentially expressed genes [file 10565_2024_9873_MOESM1_ESM.tif]

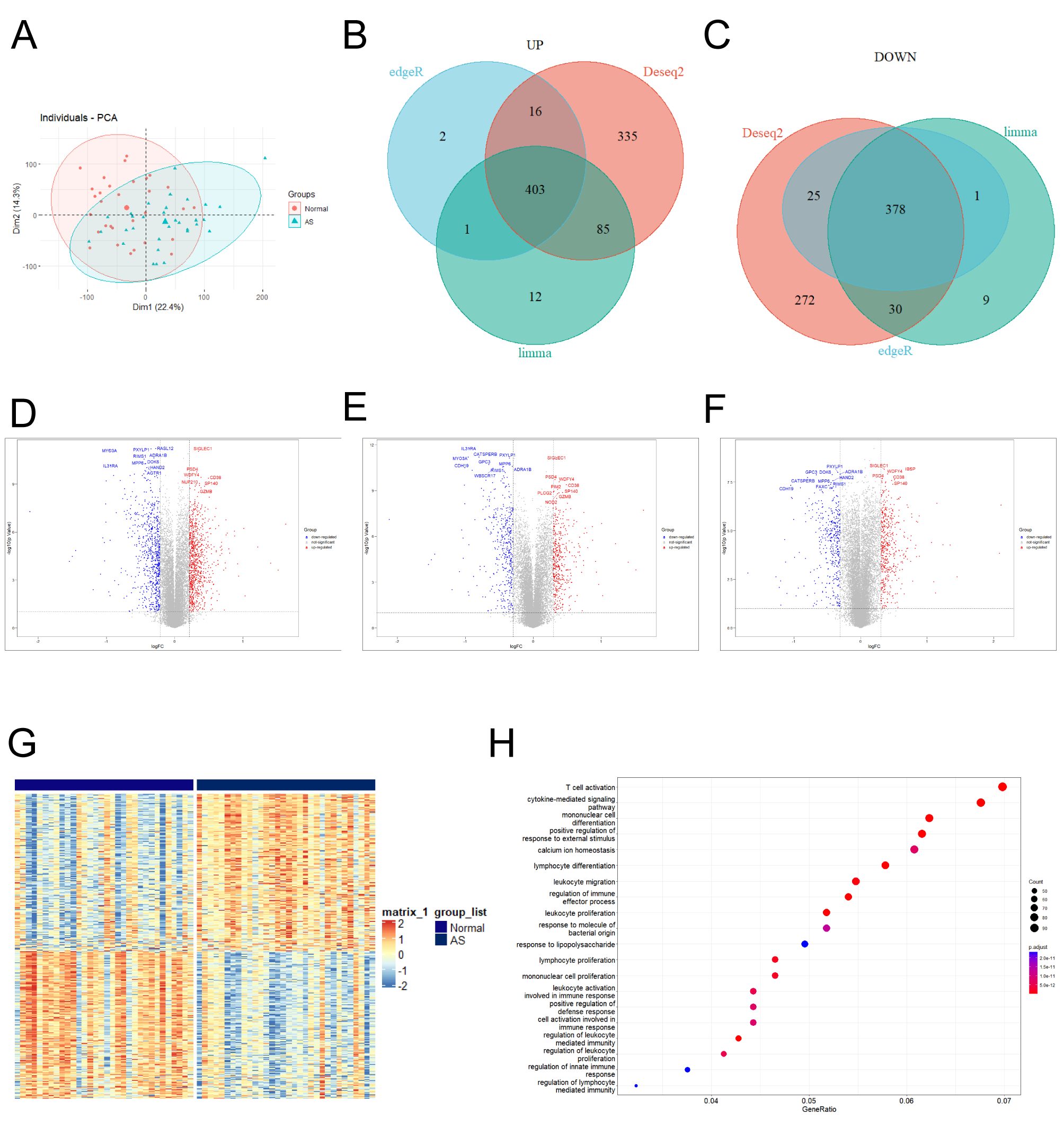

Supplement: Supplementary file 2 — Supplementary file2 (TIF 1493 KB) The results of analysis to GSE41571 (A) Feature distribution profile of early atherosclerosis genomes and advanced atherosclerosis genomes (PERMANOVA p-value < 2.8e-13 (B-C) DESeq2, edgeR, and Limma R packages were used to analyze GSE41571 datasets. The Venn diagram of highly expressed genes and lowly expressed genes were shown. (D-F) The differentially expressed analysis results of GSE41571 by DESeq2, edgeR, and Limma R packages were shown by volcanic plot. | log FC (a fold change) | = 1.0 and P < 0. 05. (G) The results of differentially expressed genes were shown by heatmap. (H) GO enrichment analysis of differentially expressed genes [file 10565_2024_9873_MOESM2_ESM.tif]

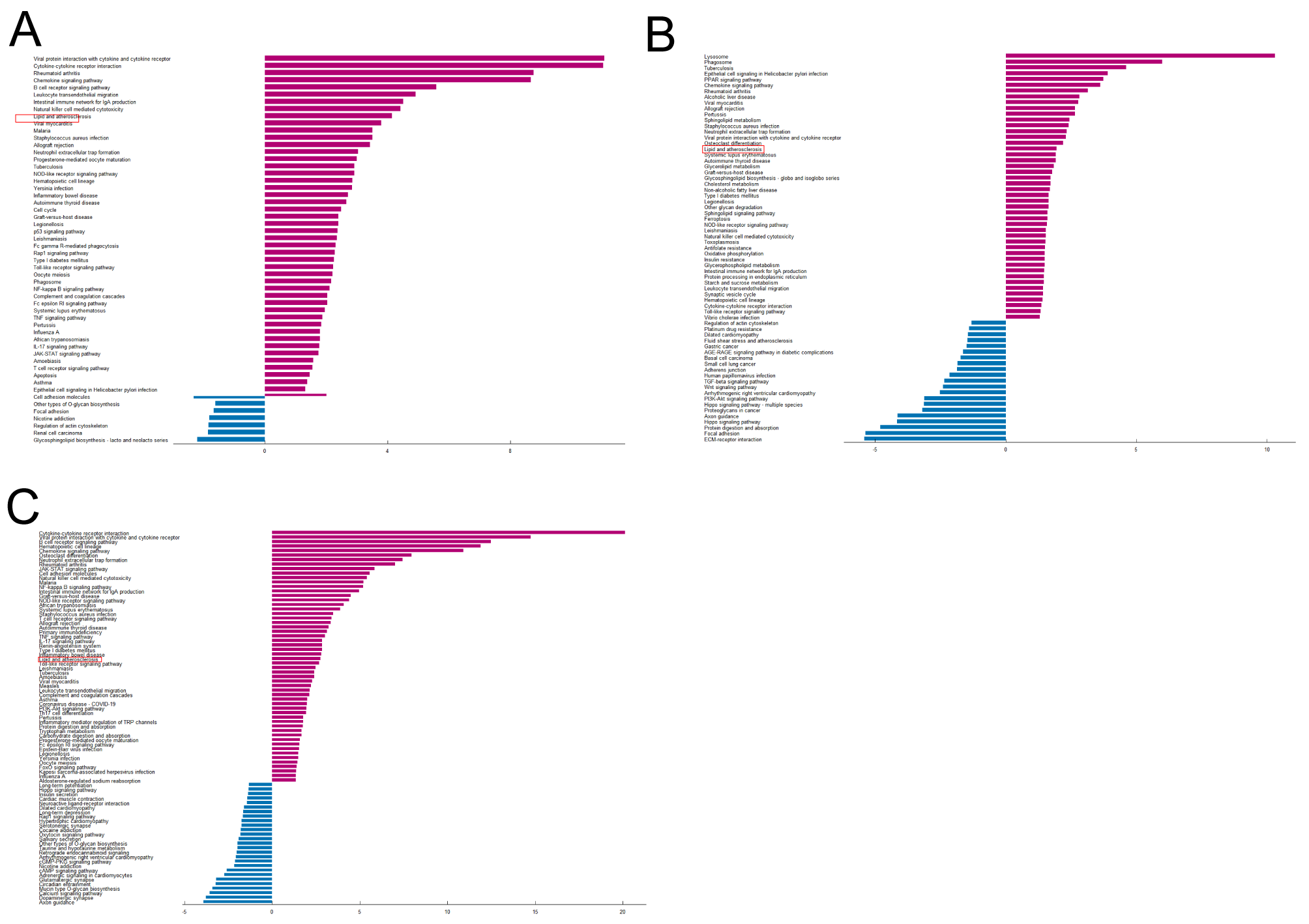

Supplement: Supplementary file 3 — Supplementary file3 (TIF 555 KB) The results of KEGG enrichment analysis of differentially expressed genes (A) KEGG enrichment analysis of differentially expressed genes in GSE28829. (B) KEGG enrichment analysis of differentially expressed genes in GSE43292. (C) KEGG enrichment analysis of differentially expressed genes in GSE41571 [file 10565_2024_9873_MOESM3_ESM.tif]

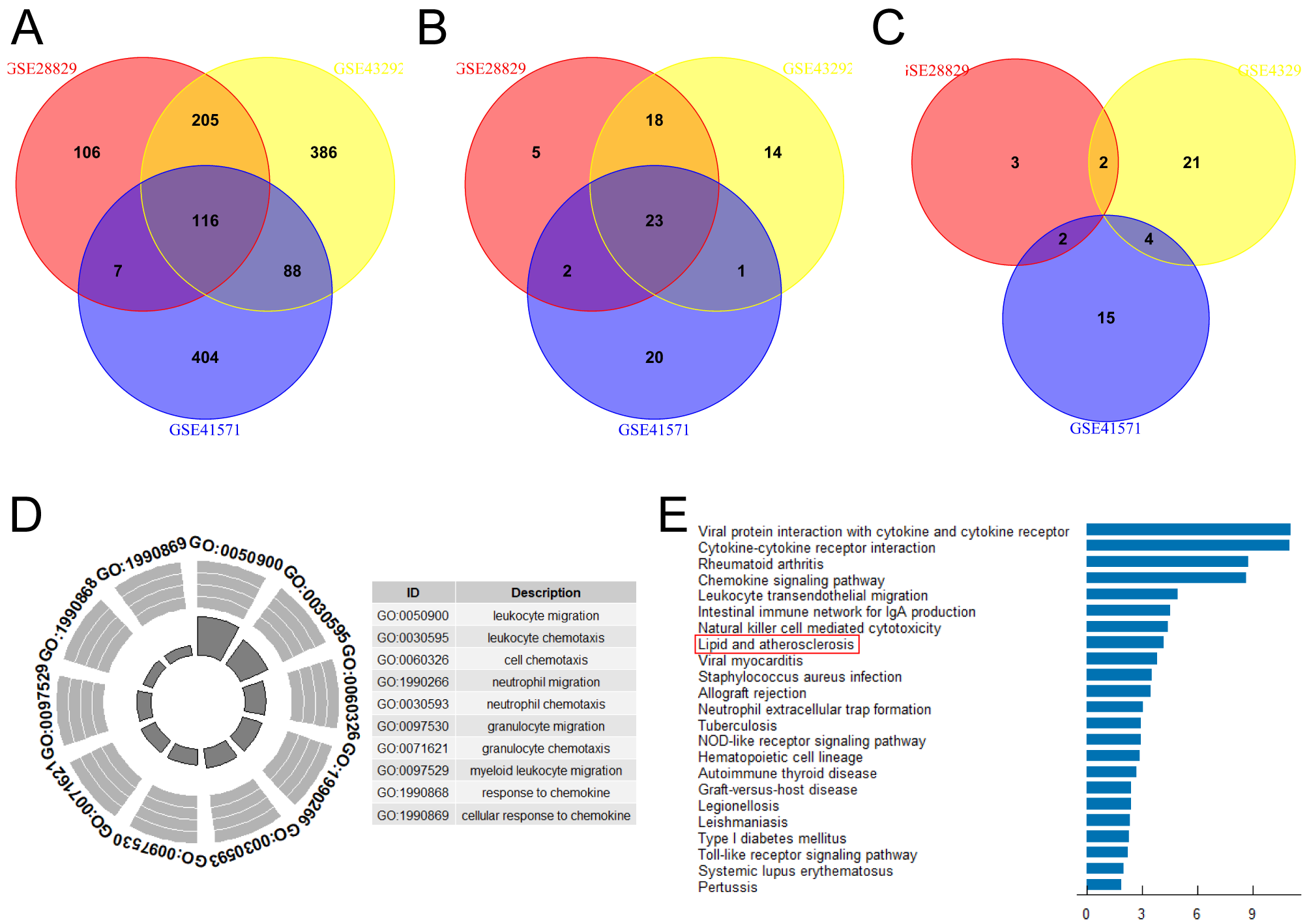

Supplement: Supplementary file 4 — Supplementary file4 (TIF 853 KB) Intersection of data set enrichment analysis and related enrichment pathways (A) The intersection of GO enrichment analysis between GSE28829, GSE43292 and GSE41571 datasets was selected. (B) The intersection of KEGG enrichment analysis to highly expressed genes between GSE28829, GSE43292 and GSE41571 datasets was selected. (C) The intersection of KEGG enrichment analysis to lowly expressed genes between GSE28829, GSE43292 and GSE41571 datasets was selected. (D) 116 signal pathway of GO enrichment analysis was shown. (E) 23 intersected KEGG enrichment analysis was shown [file 10565_2024_9873_MOESM4_ESM.tif]
